# Reward Responsiveness and Inhibition Traits Differentially Predict Economic Biases in Gain and Loss Contexts

**DOI:** 10.3389/fpsyg.2019.01948

**Published:** 2019-08-23

**Authors:** Kylie N. Fernandez, Nichole R. Lighthall

**Affiliations:** Department of Psychology, University of Central Florida, Orlando, FL, United States

**Keywords:** decision making, individual differences, economics, affect, motivation

## Abstract

Research on economic decision making has revealed specific biases in gain versus loss domains such that risky choice options are overvalued in gain conditions, implying optimism, but undervalued in loss conditions, implying pessimism. Individual differences in motivational traits and affective states have been shown to predict beliefs and behavior in risky decision making, but it is presently unclear which personal characteristics are most predictive of domain-specific biases. To address this gap in the literature, we investigated the relative influence of positive and negative motivational traits (general sensitivity to rewards and punishments) versus affective states (current levels of positive and negative emotions) on beliefs and choice behavior during a risky economic decision task. We also expanded on previous research by examining how the valence of one’s judgment context (positive context tested in Experiment 1, negative context tested in Experiment 2) may determine whether risky choice behavior is more strongly influenced by positive versus negative characteristics. Biases in belief were calculated using an economic decision task that involved estimating the value of risky “stocks” relative to safe “bonds” from experienced outcomes. Experiment 1 used a positive judgment context (likelihood of a “good stock”) while Experiment 2 used a negative judgment context (likelihood of a “bad stock”). Consistent with previous findings, we observed a domain-based bias in beliefs about stock values across experiments, such that participants exhibited optimism in gain domain and pessimism in the loss domain. Experiment 1 further revealed that domain-based bias and suboptimal choice behavior was predicted by trait-level reward sensitivity, while positive affective state (PAS) had a more limited influence on belief bias alone. Under the negative judgment context of Experiment 2, there was a similar relationship between reward sensitivity and choice behavior; however, results revealed a slightly stronger influence of negative affective state (NAS). A subsequent cross-study analysis found sensitivity to rewards was most predictive of domain-based biases. These results suggest that motivational traits – particularly those relating to reward sensitivity – are more consistent predictors of domain-based biases and risky choice behavior than affective states, but their predictive power depends the valence of the decision context.

## Introduction

Accumulating evidence indicates that contexts involving gains and losses differentially impact learning about risky economic choices and beliefs regarding their future outcomes. This difference in learning traces to the *framing effect*, where all numerical information is identical, but the description of the task presents or frames the information as either gaining or losing something of value and leads to differential error and risk sensitivity (Kahneman and Tversky, 1979; De Martino et al., 2006; Barberis, 2013; Kuhnen, 2015). For example, investor behavior after negative shocks to the stock market shows evidence of increased risk aversion and pessimism about future stock values (Todorov and Bollerslev, 2010; Bollerslev and Todorov, 2011; Guiso et al., 2018), while stock market returns are associated with greater investor optimism (Daszyńska-Żygadło et al., 2014; Lespagnol and Rouchier, 2018). Thus, losing markets appear to result in more pessimistic future beliefs about risky asset outcomes, while gaining markets result in more optimistic beliefs. Furthermore, individual levels of investor optimism lead to overvaluing assets while pessimism leads to undervaluing assets, with bias in valuation directly relating to beliefs about future returns (Lespagnol and Rouchier, 2018). In an experimental task, choices between risky “stocks” and no-risk “bonds” indicate overly pessimistic views about stocks with relatively better expected values (compared to bond) in monetary loss conditions, but overly optimistic views about stocks with relatively poorer expected values in monetary gain conditions (Kuhnen, 2015; Kuhnen and Miu, 2017). In addition, economic context manipulations that emphasize growth increase perceptions of wealth while those that emphasize scarcity increase perceptions of poverty (Millet et al., 2012). Together these findings indicate that positive and negative contexts have opposing influences on the formation of beliefs about economic choice options and their future outcomes.

Another factor that can impact the formation of beliefs is emotion. For instance, the integration of emotional information may lead to framing susceptibility or differences between loss and gain domains (De Martino et al., 2006, 2008). Additional sources indicate that decision making or judgments are derived from emotion (Baumeister et al., 2007), or an “affective heuristic”- that is, making judgments from an overall affective sense (Slovic et al., 2004). However, emotion can be separated into personal characteristics that uniquely interact with judgments (Slovic et al., 2004). Positive and negative personal characteristics such as affective states and motivational traits appear to provide an “internal context” that can influence the formation of beliefs (Baumeister et al., 2007), but it is presently unclear how such characteristics shape beliefs and which type has the greatest influence on belief formation. While both classes of personal characteristics can be considered dimensions of emotion (Harlé and Sanfey, 2010; Elliot et al., 2013), affective states are more likely to vary moment to moment and change with external conditions (Blanchette and Richards, 2010; Riepl et al., 2016; Guiso et al., 2018). Comparatively, trait-level characteristics are more enduring, consistent, and show relatively greater resistance to change (Blanchette and Richards, 2010; Dunn et al., 2010; Riepl et al., 2016). Behavioral motivations may be considered trait-like as they appear to reflect general patterns of behavior that are expressed in response to arousing contexts (Carver and White, 1994) or orienting to cues within the environment (Baumeister et al., 2007). These different temporal profiles suggest that the impact of affective states on the formation of economic biases may be more fleeting while effects of motivational traits may be more consistently observed in specific contexts over time.

Available evidence suggests that positive and negative behavioral motivations are similar to gain and loss contexts in their influence on belief formation. Positive “activation” motivations, which include reward responsiveness (sensitivity to rewarding outcomes), drive, and fun seeking, increase the likelihood of a behavior based on expected desirable outcomes in the future, while negative “inhibition” motivations (or behavioral inhibition traits) decrease the likelihood of a behavior based on expected aversive outcomes (Carver and White, 1994; Elliot et al., 2013). Specific effects of activation motivations on beliefs about risky choice options are most frequently attributed to reward responsiveness in the gain domain. For example, reward responsiveness, but not drive, has been associated with outcome expectancies for gain-associated cues in a deterministic learning task (Zinbarg and Mohlman, 1998). Additional laboratory research has shown that individuals with higher levels of reward responsiveness prefer riskier stocks under normal market conditions, indicative of greater optimism (Muehlfeld et al., 2013). Notably, preferences for risky stocks are observed in market conditions with above and below average returns (Muehlfeld et al., 2013), suggesting that reward-sensitive individuals are more optimistic about reward-associated risky choices independent of whether expected payouts are large or small. Thus, trait-level reward sensitivity appears to contribute to optimistic beliefs under gain conditions, but effects under loss conditions have not been directly examined. Studies examining the relationship between behavioral inhibition and beliefs about risky choices have yielded less consistent findings. One such study indicated that traders with high behavioral inhibition exhibited rational behavior in booming markets – enhancing their profits by increasing trading without overpaying or increasing their risk taking (Muehlfeld et al., 2013). In contrast, some findings suggest that behavioral inhibition leads to more irrational beliefs about risky choices (Desmeules et al., 2008), or indicate no relationship between behavioral inhibition and beliefs about future outcomes in either the gain or loss domain (Zinbarg and Mohlman, 1998).

Positive and negative affective states (NASs) have also been associated with distinct effects on beliefs and economic decision making. For instance, there is a general trend for people in positive moods to judge positive events as more likely (Blanchette and Richards, 2010). When choice options are associated with varying levels of reward, positive affect also leads to increased risk taking (Kuhnen and Knutson, 2011) and increased acceptance of unfair offers in the Ultimatum Game (Riepl et al., 2016). Taken together, these results indicate that positive affect leads to more positive valuing of risky and low-value choice options in the gain domain. In contrast, people in negative moods appear to judge negative events as more likely (Blanchette and Richards, 2010). In negative economic contexts such as financial crisis, negative affect may manifest as pessimism about future events and lead to risk aversion when individuals experience higher negative states, such as fear (Lerner and Keltner, 2000; Panno et al., 2015; Guiso et al., 2018). In neutral contexts, by contrast, negative affect appears to have little impact on beliefs referencing earnings forecasts, cash flow forecasts, or willingness to invest (Harding and He, 2016). Thus, available research suggests that affective states impact beliefs depending on the valence of the economic context, such that positive states increase optimism and overvaluing, while high negative states increase pessimism and undervaluing.

Despite this wealth of research, it is presently unclear whether beliefs about risky options are influenced more by state- or trait-level characteristics. The present study addresses this gap in the literature by directly comparing the influence of motivational traits and affective states on beliefs and choice behavior during risky decision making. Since previous literature often finds beliefs and behaviors to mirror each other, our predictions are that beliefs will guide behaviors (i.e., greater optimism also means selecting the risky stock option more and greater pessimism means also selecting the safe bond option more). Based on prior research, we made three specific predictions. First, we expected to find a domain-based bias across levels of affective states and motivational traits, such that beliefs about risky choice options would be more optimistic in a positive-outcome context (gain domain) but more pessimistic in a negative-outcome context (loss domain). Second, we expected that one personal characteristic type, trait-level behavioral motivations or affective states, would influence belief bias more. Finally, we manipulated both economic domain (gain, loss) and judgment context (positive estimation task, Experiment 1; negative estimation task Experiment 2) to test effects of context valence. We expected domain effects such that positive personal characteristics would contribute to optimistic economic beliefs in the gain domain and negative characteristics would contribute to pessimistic beliefs in the loss domain. Evidence supporting this hypothesis has been reported for both motivational traits and affective states, but findings have been less consistent for trait-level behavioral inhibition relative to reward sensitivity and affective states. Although the majority of related studies examined effects of motivational traits and affective states in valence-congruent economic contexts (e.g., reward sensitivity in a gain maximizing task, negative affect in a financial crisis), there is some suggestion that effects personal characteristics depend on the valence of one’s context. We expect our inclusion of judgment context (positive judgment context, Experiment 1; negative judgment context Experiment 2) to expand current economic valence context effects.

## Experiment 1

Experiment 1 used an economic decision-making task with separate gain and loss outcome domains to investigate beliefs and choice behavior related to risky choice options. Behavior was examined in a positive judgment context, such that participants were asked to estimate the likelihood that the current risky option yielded dividends from a “good” (i.e., optimal payout) distribution. Primary analyses examined trait-level behavioral motivation and state-level affect as potential predictors of estimation bias and choice behavior by economic domain.

### Materials and Methods

#### Participants

The study included an ethnically diverse sample of 92 college students who were fluent in English and had normal or corrected-to-normal vision. Our sample size minimum was based on that of Kuhnen (2015) and our experimental task is a modified version of that paradigm (complies with Simmons et al., 2011). Three participants were excluded due to technical errors. The final sample included 89 participants (35 male) ages 18 – 28 (*M* = 19.12, *SD* = 1.72; see Table 1 for race and ethnicity information). Participants were monetarily compensated with a minimum of $10 and the possible addition of a cash bonus (total payout range: $11.20–24.00). This study was carried out in accordance with the recommendations of the University of Central Florida’s Institutional Review Board with verbal informed consent from all subjects. The Institutional Review Board determined the study was minimal risk and therefore did not require written informed consent. All subjects gave verbal informed consent in accordance with the Declaration of Helsinki. The protocol was approved by the Institutional Review Board.

**TABLE 1:** Race and ethnicity for participants in Experiment 1 and Experiment 2.

	**Exp. 1**	**Exp. 2**	**Total**
Race			
African-American	16%	18%	17%
Asian	3%	8%	6%
Caucasian	64%	54%	59%
Pacific Islander/Native Hawaiian	1%	1%	1%
Multi-racial/Other	14%	13%	13%
Prefer not to answer	2%	6%	4%
Ethnicity			
Hispanic/Latino	24%	28%	26%
Non-Hispanic/Latino	75%	71%	73%
Prefer not to answer	1%	1%	1%

#### Procedure

Prior to the experimental task, participants completed demographic and psychosocial questionnaires, as well as an executive functioning task. Individual differences in affective states were measured using the positive affective state (PAS) and NAS subscales of the Positive Affect and Negative Affect Schedule (PANAS; Watson et al., 1988). Comparable trait measures were selected from two subscales of the Behavioral Inhibition/Behavioral Activation Scale (BIS/BAS; Carver and White, 1994). Specifically, trait-level sensitivity to aversive outcomes was assessed with the BIS subscale (Carver and White, 1994). Trait-level sensitivity to rewarding outcomes was assessed with the BAS reward responsiveness (BAS-RR) subscale, which is the most direct measure of sensitivity to positive experiences of the three BAS subscales. At the end of the session, participants completed additional post-experimental, financial literacy, and sleep quality questionnaires. Data for the executive function task and additional non-demographic questionnaires are not presented here.

#### Economic Decision Task

**FIGURE 1:**
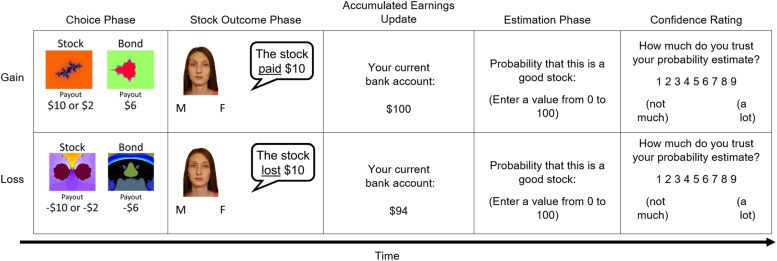
Sequence of the phases within a single trial in the economic investment task. The individual depicted provided written informed consent for the publication of this image.

The experimental task is a modified version of the Active Task from Kuhnen (2015). Participants first received instructions and then performed four practice trials (2 in each domain type), which were structured exactly like experimental trials. To provide additional clarification about the task, participants were able to ask questions and review task instructions during the practice trials and were given the opportunity to repeat the practice trials once. Instructions described possible stock and bond payouts, the number of trials in each block, and the two possible payout distributions for stocks. The task included 12 trial blocks that were pseudorandomized under two conditions: gain and loss, which consisted of 6 individual trials or 36 trials in each domain. In the gain condition, choice options resulted in positive earnings of varying magnitude and optimal behavior maximized those earnings. In the loss condition, choice options resulted in losses of varying magnitude and optimal behavior minimized those losses. Across domain conditions, each trial included the following phases: choice, stock outcome, accumulated earnings update, estimation, and confidence rating (Figure 1).

On each self-paced choice phase, participants were asked to choose between a safe security (“bond”) and a risky security (“stock”). Stock dividend magnitudes were either high or low. Gain stock payoffs were either + $10 or + $2 and loss stock payoffs were either −$10 or −$2. Bonds had static payouts of + $6 in the gain condition and −$6 in the loss condition. Stocks drew their outcomes from either a “good” or “bad” distribution. Stocks in the good distribution had a 70% chance of high dividend payouts (gain: + $10; loss: −$2), whereas stocks in the bad distribution had a 70% chance of low dividend payouts (gain: + $2; loss: −$10). Participants were informed of the different distributions and differences between stock and bond choices during the instruction period (see section “Investment Task Instructions” in Supplementary Material). Gain and loss blocks had an equal number of good and bad payout distributions. The order of payout distribution conditions was pseudorandomly assigned at the beginning of the experiment.

After each choice, the self-paced outcome phase included a “stockbroker” (trial-unique face image; face stimuli from Minear and Park, 2004) reporting the stock payoff. Critically, stock payoffs were presented whether participants selected the stock or bond, thereby requiring the updating of beliefs about risky choice options on each trial. As described above, the stock-outcome phase required participants to indicate the gender of the stockbroker face using a key press. Following the outcome phase, participants were presented with a tally of their accumulated earnings from the payouts of their past choices for 2 s. To assess biases in belief, participants were asked to estimate the likelihood that the stock comes from the good distribution as a percentage from 0 to 100 based on experienced payout history in the current block. Each block included six trials; thus, participants updated their beliefs about each stock’s outcome probability six times. The last self-paced trial phase asked participants to provide a rating of trust in their probability estimate from 1 (not much) to 9 (a lot; confidence data not included here).

Our primary outcome measure of valenced estimation error was calculated from the difference between the participant’s probability estimation and the objective Bayesian outcome probability that the stock was from the good payout distribution on a given trial (i.e., subjective – objective probability). Objective probabilities were based on each stock’s current history of optimal versus non-optimal payouts (see section “Objective Probability Calculation” in Supplementary Material and Supplementary Table S1). Thus, higher valenced estimation errors represented overestimations that the stock was good (optimistic beliefs) and lower valenced estimation errors represented underestimations that the stock was good (pessimistic beliefs). It is important to note that the valenced error specifically means the direction of participant’s error has meaning, and that is why “valenced error” is the terminology used for this study. Subject-level averages were then calculated from trial-wise values within domain conditions (gain, loss) and used to determine an overall estimation error score (i.e., average of valenced error for gains – average of valenced error for losses).

Our outcome measure of optimal choice behavior reflected the proportion of choices that aligned with the objective probability that the current stock was from the good distribution. Stock selections were coded as optimal when the objective probability that the stock was good was above 50%. Bond selections were coded as optimal when the objective probability that the stock was good was below 50%. Trials with objective probabilities of exactly 50% were excluded from analyses of optimal choice behavior. Separate optimal choice measures were calculated for behavior in the gain domain, loss domain, and across domain conditions. In this way, optimal choice behavior also represented beliefs about choice-option values.

Two types of monetary incentives were included in the task to encourage optimal choice behavior and accurate probability estimations. Specifically, participants were given 10% of their total accumulated earnings from the payouts of their stock/bond choices in addition to $0.10 for each stock probability estimation that was within 5% of the objective probability. At the start of the task, participants were endowed with $15 in their “bank account” and negative total earnings resulted in losses from that endowment. All participants earned a bonus for their performance (lowest final accumulated total earnings = $11.20).

#### Statistical Analyses

Statistical analyses were conducted using SPSS 25 (SPSS, RRID:SCR_002865). Individual difference measures included positive and negative indices of affective states (PAS, NAS) and motivational traits (BAS-RR, BIS). Preliminary analyses included Spearman’s rho rank correlations (Supplementary Table S2), since not all of the data met the normality assumption required for Pearson correlations but did meet the assumptions of variable type and monotonic relationship. In addition, one sample *t*-tests were conducted only for valenced estimation error to check for domain-based bias (Supplementary Table S3). Our primary model comparisons investigated relationships between the individual difference measures and the dependent variables of interest: averages for overall valenced error (i.e., signed difference between subjective and objective probabilities) and optimal choice behavior. Secondary models examined valenced error and optimal choice behavior separately in the gain domain and the loss domain. To determine if observed individual difference effects on risk taking drove group differences in optimal choice, we conducted *post hoc* tests of effects on stock selection in gain versus loss domains (i.e., risk taking bias) and within domain conditions. Specifically, risk taking was measured by the frequency of stock selections on the first trial of each new block when stock and bond options had equivalent outcome probabilities and no associated outcome history. The four personal characteristic scales were included in two multiple linear regression models to determine predictors of valenced error and optimal choice behavior (one trait model included BAS-RR and BIS as predictors of the dependent variable and the state model included PAS and NAS as predictors of the dependent variable. One trait model and one state model is necessary to offset the multicollinearity assumption, since rank order correlations and logic indicate that current affective state-levels are related to more general motivational trait-levels (Supplementary Table S2). To ensure that the models met the proper assumptions, we verified that the data has a linear relationship, multivariate normality (the data has normally distributed residuals), have no evidence of autocorrelation (Durbin–Watson testing for all models fell within the range of 1 to 3), and to further avoid the problem of multicollinearity (Cohen et al., 2003) we used subscales designed to be independent of each other (Carver and White, 1994; Watson et al., 1988). In addition, all VIF values were greater than 0.1 and tolerance valences were less than 2.5. Furthermore, the assumption of homoscedasticity was investigated using Breusch–Pagan tests (Cohen et al., 2003). If heteroscedasticity was identified by Breusch–Pagan (Pryce, 2002), the potential impact on model interpretation was determined; no models were determined to have a large heteroscedasticity magnitude to warrant (protocol and thresholds come from Cohen et al., 2003). All models met the statistical assumptions for testing unless noted otherwise. Reported results include adjusted R squared, and standardized coefficients, unstandardized coefficients, and significance levels. It is important to note that the dependent variables were constrained by the possible responses themselves (see Supplementary Table S2 for more details). Potential outliers needed to meet criteria to be excluded including a Cook’s Distance of at least 1 and residuals larger than ± 3.0 (thresholds come from Cohen et al., 2003). No such outliers were identified and thus all participants are included in the following analysis (complies with Simmons et al., 2011).

### Results

#### Valenced Estimation Error

Correlation results indicated that BAS-RR predicted overall estimation error such that higher trait-level reward sensitivity predicted greater domain-based bias, while PAS predicted more positive estimation errors in the gain domain (see Supplementary Table S2). For the one sample *t*-tests, overall valenced error reflected relatively overvaluing and thus greater optimism in the gain domain and undervaluing or greater pessimism in the loss domain (see Supplementary Table S3). These results support previous literature findings of domain-based bias.

**FIGURE 2:**
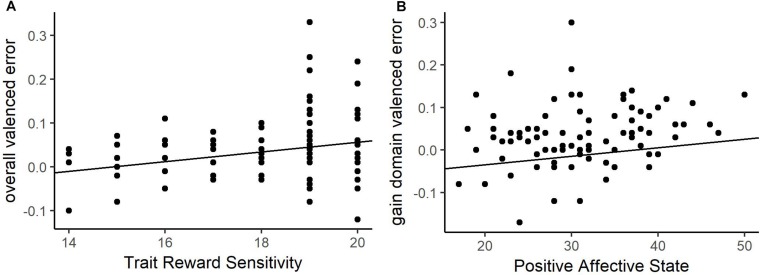
Valenced estimation error relationships in the positive judgment context for overall estimation error and trait-level reward responsiveness **(A)**, estimation error in the gain domain and positive affective state (PAS) **(B)**. Higher levels of reward sensitivity, and positive affect predict higher overall valenced error, or optimism.

The regression analysis of overall valenced estimation error revealed a significant predictor in the trait model but none were found in the state model (see Table 2 for details). BAS-RR was the only significant predictor when estimating the likelihood of having a good stock. As shown in Figure 2A, higher trait-level reward sensitivity was associated with more positive bias in beliefs, or optimism. To determine if this effect was driven by estimation errors in a single domain, we separately examined valenced estimation error on gain and loss blocks. The secondary models for overall valenced estimation error found no significant predictors in the loss domain, but found one significant predictor in the gain domain for the state model which was PAS. PAS score increases related to more overvaluing of stock estimations, or optimism (Figure 2B). However, the restriction of PAS predicting estimation error in the gain domain in a positive judgment context suggests it is limited in its utility as a general predictor of estimation error. Reward sensitivity is therefore a better predictor of estimation error, as it is a composite of both domains and has a higher standardized coefficient. In general, the results suggest when assessing the likelihood that a risky option will yield optimal outcomes, individuals with greater reward sensitivity make estimation errors that align with the valence of choice outcomes. That is, when payouts are positive, they overestimate the likelihood of optimal outcomes (large gains), but when payouts are negative, they underestimate the likelihood of optimal outcomes (small losses).

**TABLE 2:** Model statistics for the multiple linear regressions in Experiment 1 by dependent variable and then model type (trait or state); includes the predictor variable information to the right of the model information.

**Model**	***F***	***df***	**Adj *R*^2^**	**Model *p*-value**	**Predictor**	***B***	**β**	**Predictor *p*-value**
Overall Error								
**Trait**	**3.444^∗^**	**2, 86**	**0.053^∗^**	**0.036**	**BAS-RR**	**0.011**	**0.264^∗^**	**0.014^∗^**
					BIS	0.001	0.035	0.743
State	1.678	2, 86	0.015	0.193	PAS	0.002	0.194	0.071
					NAS	0.001	0.034	0.753
Gain Error								
Trait	0.822	2, 86	−0.004	0.443	BAS-RR	0.005	0.136	0.211
					BIS	−0.001	−0.04	0.972
State	2.789	2, 86	0.039	0.067	**PAS**	**0.002**	**0.233^∗^**	**0.029^∗^**
					NAS	0.003	0.108	0.308
Loss Error								
Trait	1.711	2, 86	0.016	0.187	BAS-RR	−0.006	−0.157	0.147
					BIS	−0.002	−0.094	0.386
State	0.290	2, 86	−0.016	0.749	PAS	0.000	0.030	0.779
					NAS	0.002	0.079	0.466
Overall Choice								
**Trait**	**3.866^∗^**	**2, 86**	**0.061^∗^**	**0.025**	**BAS-RR**	**−0.026**	**−0.288^∗∗^**	**0.007^∗∗^**
					BIS	0.004	0.094	0.374
State	0.111	2, 86	−0.021	0.895	PAS	0.001	0.043	0.691
					NAS	0.002	0.031	0.773
Gain Choice								
**Trait**	**3.936^∗^**	**2, 86**	**0.063**	**0.023**	**BAS-RR**	**−0.028**	**−0.294^∗∗^**	**0.006^∗∗^**
					BIS	0.003	0.058	0.581
State	0.022	2, 86	−0.023	0.978	PAS	0.000	0.013	0.906
					NAS	0.001	0.020	0.853
Loss Choice								
Trait	2.859	2, 86	0.041	0.063	**BAS-RR**	**−0.023**	**−0.239^∗^**	**0.027^∗^**
					BIS	0.006	0.122	0.251
State	0.224	2, 86	0.005	0.800	PAS	0.002	0.068	0.529
					NAS	0.002	0.031	0.777

*^∗^ indicates significance at *p* < 0.05 while ^∗∗^ indicates significance at *p* < 0.01.*

#### Optimal Choice Behavior

Correlation analyses indicated that BAS-RR predicted choice behavior in both domains and overall, such that higher reward sensitivity predicted lower optimal choice performance (see Supplementary Table S2). BAS-RR was the only predictor of choice behavior identified by the correlations.

**FIGURE 3:**
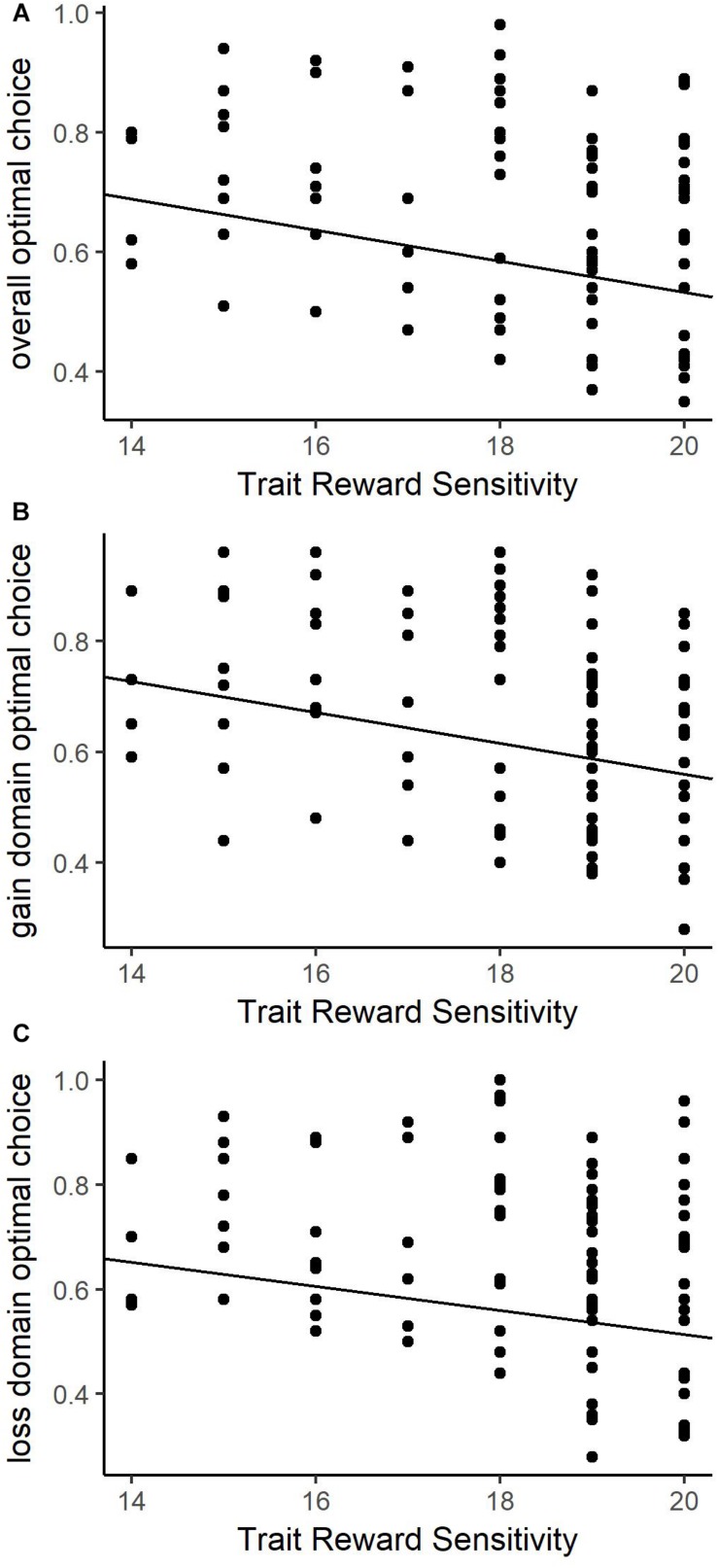
Choice behavior and trait-level reward responsiveness relationships in the positive judgment context for total suboptimal choice behavior **(A)**, suboptimal choice behavior in the gain domain **(B)**, and suboptimal choice behavior the loss domain **(C)**. Higher levels of reward sensitivity predict greater suboptimal choice proportion for all contexts.

Overall optimal behavior was predicted only by BAS-RR in the trait model and no predictors in the state model (see Table 2 for details). Figure 3A demonstrates that as reward sensitivity decreases, the proportion of optimal choices increases. Secondary models also revealed significant BAS-RR relationship with optimal choice behavior in the trait model for gain and loss domains, but not state model predictors or BIS. Both secondary models were consistent with the primary model: decreasing reward sensitivity relates to an increase in optimal choice (Figures 3B,C). These parallel domain-specific results illustrate consistent behavioral differences based on trait-level reward sensitivity. Therefore, optimal choice behavior was significantly greater in individuals with lower reward sensitivity in both domains.

To determine whether observed effects of individual difference measures on optimal choice was driven by effects on risk taking, we investigated risky choice bias as measured by frequency of stock selections on the first trial of each block for gain versus loss conditions (i.e., trials with equivalent outcome probabilities for stock and bond options). Initial correlation results indicated that risk taking would predict overall optimal choice behavior such that decreased risk taking leads to increased optimal choice behavior (see Supplementary Table S2). However, when we conducted a *post hoc* analysis, it yielded no significant predictors of overall risk taking bias (*p* > 0.30 for all scales; *p* > 0.53 for all models). Risk taking also did not differ by states or traits when risk taking was separately examined in the gain condition (*p* > 0.33 for all scales; *p* > 0.59 for all models) or loss condition (*p* > 0.48 for all scales; *p* > 0.74 for all models).

### Discussion

Together, findings from Experiment 1 illustrate consistent differences in economic beliefs based on trait-level reward sensitivity. Specifically, individuals with greater reward sensitivity were more influenced by the valence of risky-choice outcomes, exhibiting overly optimistic beliefs about options that led to gains and overly pessimistic beliefs about options that led to losses. Results also revealed a selective, but weaker effect of state-level positive affect on beliefs, such that individuals with lower positive affect had lower estimation errors in the gain domain. Results for choice behavior were consistent with higher reward sensitivity relating to less accurate beliefs about stock probabilities. Specifically, we found that individuals with higher reward sensitivity were less likely to make optimal choices than those with lower reward sensitivity and this effect was not explained by differences in risk taking behavior. Together, findings from Experiment 1 indicated that trait-level reward sensitivity was a more robust predictor of biased beliefs and choice behavior compared with positive affect. However, Experiment 1 only tested the relationship between personal characteristics and belief formation in a positive judgment context (i.e., judgments about the likelihood of having the “good” stock). We conducted a second experiment in a negative judgment context (likelihood of having the “bad” stock) to examine the robustness of our observed findings and address the possibility that the relationship between personal characteristics and beliefs depends on the valence of the judgment context.

## Experiment 2

The results of Experiment 1 suggested that the motivational trait of reward sensitivity was the strongest predictor of domain-based bias in economic beliefs and behavior, but these effects were restricted to a positive judgment context (i.e., positive estimation task framing). To examine the generalizability and boundary conditions of this finding, Experiment 2 included a modification to the economic task implemented in Experiment 1. Specifically, the economic decision task in Experiment 2 asked participants to estimate the likelihood that the current risky option yielded dividends from a “bad” (i.e., non-optimal payout) distribution. All other models either met the original homoscedasticity or were determined to have heteroscedasticity effects that did not impact the interpretation of the models (Cohen et al., 2003).

### Materials and Method*s*

#### Participants

The study included a diverse sample of 110 college students, who were fluent in English and had normal or corrected-to-normal vision. Seven participants were excluded due to technical errors. The final sample included 103 participants (32 male) ages 18–29 (*M* = 19.68, *SD* = 2.43; see Table 1 for race and ethnicity information). Participants were monetarily compensated with a minimum of $10 and the possible addition of a cash bonus (total payout range: $10.00–24.50). This study was carried out in accordance with the recommendations of the University of Central Florida’s Institutional Review Board with verbal informed consent from all subjects. The Institutional Review Board determined the study was minimal risk and therefore did not require written informed consent. All subjects gave verbal informed consent in accordance with the Declaration of Helsinki. The protocol was approved by the Institutional Review Board.

#### Procedure

Procedures were the same as in Experiment 1, with one exception: during the economic decision-making task participants were asked to determine the likelihood that the current stock was a “bad stock” (i.e., paid from the bad distribution). Language used in the instructions, practice trials, and estimation phase reflected this change.

#### Statistical Analyses

To facilitate cross-study result comparisons, responses in the estimation phase were transformed to match those in Experiment 1. That is, Experiment 2 asked participants to determine how “bad” the stock was but estimations and objective probabilities were reversed to reflect probabilities of a “good” stock (positive judgment context). Otherwise, analyses were identical to Experiment 1.

### Results

#### Valenced Estimation Error

Correlation results demonstrated that BIS predicted overall estimation error such that higher trait-level reward sensitivity predicted greater domain-based bias (see Supplementary Table S2). For the one sample *t*-tests, the error in the gain domain was marginal, however, the direction of the error supports overvaluing stocks in the gain domain. Therefore, the pattern continued where overall valenced error reflected relatively overvaluing and thus greater optimism in the gain domain and undervaluing or greater pessimism in the loss domain (see Supplementary Table S4). These results support previous literature findings of domain-based bias.

**FIGURE 4:**
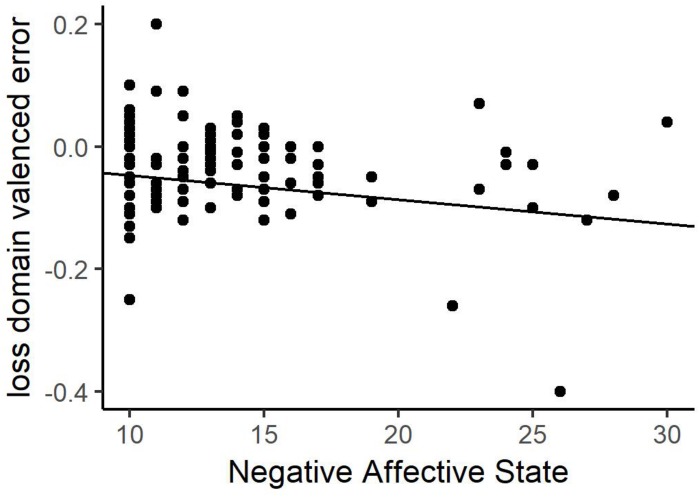
Valenced estimation error relationships with negative affective states (NAS) in the negative judgment context for the loss domain. Higher levels negative affect predict greater error in the loss domain.

Regression analysis of overall valenced estimation did not identify any significant predictors (see Table 3 for details) for either primary model. Secondary models separately examined valenced estimation error in gain and loss blocks. The gain domain found no significant predictors for either model. For the loss domain, a significant predictor of NAS was identified in the state model only. Therefore, we observed no consistent indicator of errors in belief under a negative judgment context across domains. However, in the loss domain, increasing negative affective scores lead to lower estimations, or increased pessimism (see Figure 4). Consistent with Experiment 1, affective states only predict errors in beliefs in the domain that matches the judgment context. In Experiment 1, PAS was predictive in the gain domain under the positive judgment context; in Experiment 2, NAS is predictive in the loss domain under negative judgment context.

**TABLE 3:** Model statistics for the multiple linear regressions in Experiment 2 by dependent variable and then model type (trait or state); includes the predictor variable information to the right of the model information.

**Model**	***F***	***df***	**Adj R^2^**	**Model *p*-value**	**Predictor**	***B***	**β**	**Predictor *p*-value**
Overall Error								
Trait	1.869	2, 100	0.017	0.160	BAS-RR	0.003	0.05	0.641
					BIS	0.005	0.18	0.085
State	0.731	2, 100	−0.005	0.484	PAS	0.001	0.045	0.654
					NAS	0.002	0.104	0.302
Gain Error								
Trait	0.281	2, 100	−0.014	0.755	BAS-RR	0.002	0.036	0.723
					BIS	0.001	0.058	0.568
State	1.296	2, 100	0.006	0.278	PAS	0.001	0.137	0.172
					NAS	−0.002	−0.104	0.298
Loss Error								
Trait	1.575	2, 100	0.011	0.212	BAS-RR	−0.001	−0.024	0.809
					BIS	−0.003	−0.168	0.098
**State**	**3.122^∗^**	**2, 100**	**0.040**	**0.048**	PAS	0.001	0.080	0.416
					**NAS**	**−0.004**	**−0.242^∗^**	**0.016**
Overall Choice								
**Trait**	**3.727^∗^**	**2, 100**	**0.051**	**0.027**	**BAS-RR**	**−0.028**	**−0.259^∗^**	**0.010**
					BIS	0.001	−0.017	0.867
**State**	**5.471^∗∗^**	**2, 100**	**0.081**	0.006	PAS	−0.003	−0.120	0.216
					**NAS**	**−0.010**	**−0.272^∗∗^**	**0.006**
Gain Choice								
**Trait**	**4.463**	**2, 100**	**0.064^∗^**	**0.014**	**BAS-RR**	**−0.030**	**−0.250^∗^**	**0.012**
					BIS	−0.005	−0.097	0.322
**State**	**7.076**	**2, 100**	**0.106^∗∗^**	**0.001**	PAS	−0.004	−0.175	0.067
					**NAS**	**−0.011**	**−0.279^∗∗^**	**0.004**
Loss Choice								
Trait	2.168	2, 100	0.031	0.078	**BAS-RR**	**−0.027**	**−0.227^∗^**	**0.025**
					BIS	0.003	0.069	0.492
State	2.834	2, 100	0.035	0.064	PAS	−0.001	−0.048	0.627
					**NAS**	**−0.009**	**−0.219^∗^**	**0.028**

*^∗^ indicates significance at *p* < 0.05 while ^∗∗^ indicates significance at *p* < 0.01.*

#### Optimal Choice Behavior

Correlation results indicated that BAS-RR predicted overall choice behavior and choice behavior in the gain domain, such that higher reward sensitivity predicts more suboptimal choice behavior, while increased PAS predicted more suboptimal choice behavior in the gain domain (Supplementary Table S2). Furthermore, NAS predicted overall choice behavior and choice behavior in both domains where increased NAS predicted more suboptimal choice behavior.

**FIGURE 5:**
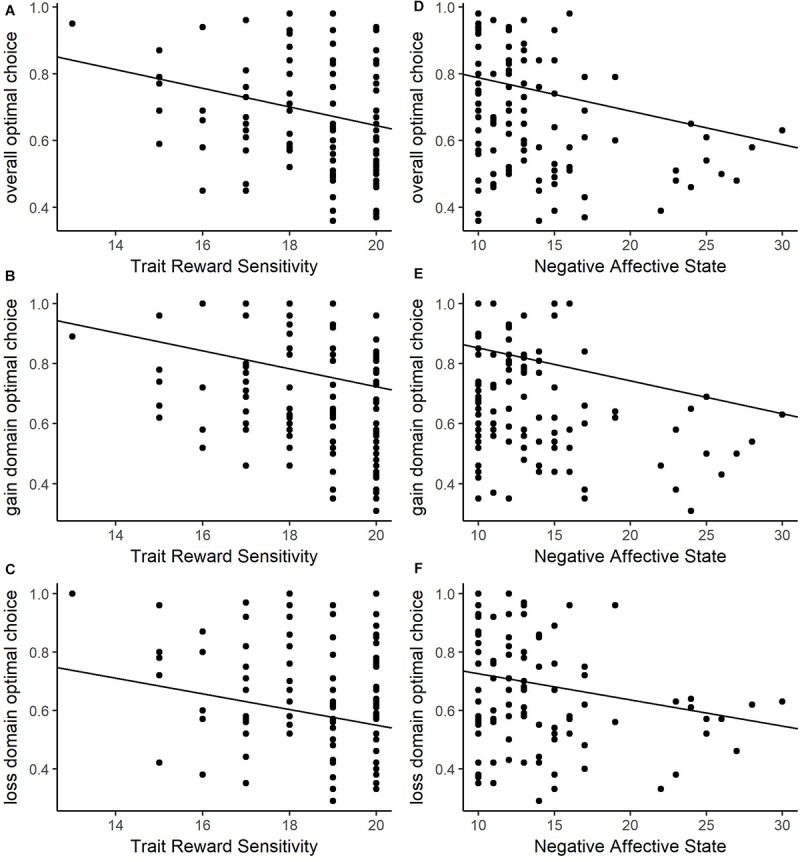
Choice behavior relationships in the negative judgment context for total suboptimal choice behavior and trait-level reward responsiveness **(A)**, suboptimal choice behavior in the gain domain and trait-level reward responsiveness **(B)**, and suboptimal choice behavior the loss domain and trait-level reward responsiveness **(C)** total suboptimal choice behavior and state-level negative affect **(D)**, suboptimal choice behavior in the gain domain and state-level negative affect **(E)**, and suboptimal choice behavior the loss domain and state-level negative affect **(F)**. Higher levels of reward sensitivity and negative affect predict greater suboptimal choice proportion for all contexts.

Optimal choice behavior in general was predicted by BAS-RR from the trait model and NAS from the state model (see Table 3 for details). Consistent with Experiment 1, decreasing reward sensitivity related to an increase in optimal choice proportion (Figures 5A–C). Unique to Experiment 2 is the relationship between decreasing NAS scores and higher optimal choice proportion (Figures 5D–F). To determine if either relationship was driven by choice behavior in a single domain, we separately examined choice behavior in gain and loss blocks. Both secondary models mirrored the primary model findings: in the gain domain, BAS-RR from the trait model and NAS from the state model were significant. In the loss domain, BAS-RR from the trait model and NAS from the state model again were significant. For all conditions, higher optimal choice behavior related to decreased reward sensitivity and lower NAS scores. Based on the findings in Experiment 1, reward sensitivity is the most consistent predictor of choice behavior in a negative judgment context. However, the predictive ability of NAS is compelling in its own right.

Additionally, correlations for risk taking did not find any significant relationships (Supplementary Table S2). As in Experiment 1, *post hoc* analysis of risky choice bias yielded no significant predictors of overall risk taking bias in a negative judgment context (*p* > 0.28 all scales). Furthermore, risk taking did not differ by states or traits when risk taking was separately examined in the gain condition (*p* > 0.28 all scales; *p* > 0.28 all models) or loss condition (*p* > 0.14 all scales; *p* > 0.32 all models).

### Discussion

Consistent with the findings from Exp. 1, results from Exp. 2 indicated choice behavior is related to reward sensitivity, such that lower sensitivity leads to increased optimal choice behavior. Regression results in Exp. 2 did not find BIS to be a significant predictor of valenced estimation error; however, it was indicated as a predictor in the correlation analysis. Therefore, BIS may exert a relatively weak effect on beliefs about risky choices in a negative judgment context. Experiment 2 results differ from Experiment 1 since NAS is an additional predictor of choice behavior, regardless of domain. Also consistent with Experiment 1, higher affective state scores predict specific domain biases in belief. Specifically, for Experiment 2, which is the negative judgment context, NAS was predictive of belief in the loss domain. These findings suggest the effects of trait reward sensitivity are consistent, but that the strength of its predictive power is dependent on the valence of the economic judgment context.

## Cross-Study Analysis

We conducted a cross-study analysis to directly compare effects of group-level predictors under positive (Experiment 1) and negative (Experiment 2) judgment contexts. This analysis was conducted in order to determine whether predictors of belief and choice behavior were consistent across studies or depended on the valence of the estimation task (i.e., probability estimations for “good” versus “bad” stocks).

### Materials and Method*s*

#### Statistical Analyses

Cross-study analyses examined significant predictors of valenced estimation error and optimal choice from Experiments 1 and 2, as well as main effects and interactions with judgment context (Experiment 1/positive, Experiment 2/negative). Thus, for valenced estimation error, the cross-study analysis tested main effects of BAS-RR, and study, as well as the interaction between experiment judgment context positive motivational traits. For optimal choice behavior, the cross-study analysis tested main effects of BAS-RR, and NAS, as well as interactions between experiment judgment context and these state/trait subscales. As significant predictors of risk taking were not observed in either study, this variable was not included in the cross-study analysis. Due to failed assumptions testing that could not be corrected, linear regression models and their interpretations are extremely limited. Limitations are noted below (for details see Supplementary Tables S5–S7).

### Results

#### Valenced Estimation Error

Results of the overall valenced estimation error model revealed a significant predictor of trait-level reward sensitivity. However, as a result of the multicollinearity, the extent of a relationship between experiment and reward sensitivity does not exist or is difficult to determine from the current model. Additional interpretation beyond that scope is left for future studies.

#### Optimal Choice Behavior

Analyses indicates that average optimal choice behavior is predicted by reward sensitivity, but relationships are unclear between experiment and the interaction. Multicollinearity means it is difficult to clarify those relationships further. For the state model, effects of NAS appear to rely on the interaction of experiment and NAS, since the interaction term appears meaningful, but the main effect term for negative state does not. This provides support for some effect of judgment context. Additional interpretation beyond that scope is left for future studies.

### Discussion

The cross-study findings suggest possible impacts of experiment, and therefore judgment context, with personal characteristics may exist. However, due to structural multicollinearity, it is difficult to determine the exact interactions and effects that exist. We therefore conclude that our main personal characteristic findings seem supported, and leave additional investigation to the future.

## General Discussion

Prior findings from real-world financial investments and laboratory experiments have suggested that affective states and motivational traits influence our beliefs about risky decision options and related choices (Lerner and Keltner, 2000; Scheres and Sanfey, 2006; Muehlfeld et al., 2013; Riepl et al., 2016). Both classes of personal characteristics have positive and negative dimensions that have been associated with optimistic and pessimistic beliefs, respectively; however, the relative impact of affective states versus motivational traits on belief formation has never been directly examined. The present studies sought to address this gap in the literature, and to determine how effects of personal characteristics may differentially impact the formation of beliefs in positive and negative judgment contexts. Our studies yielded three primary findings. First, consistent with previous research, we observed a domain-based bias such that beliefs about risky options were overly optimistic when learning occurred in the gain domain (positive outcomes) but overly pessimistic when learning occurred in the loss domain (negative outcomes; Kahneman and Tversky, 1979; Kuhnen, 2015; Kuhnen and Miu, 2017). Second, we found that positive motivational traits were consistent predictors of domain-based biases and associated choice behavior especially for positive judgment contexts. Finally, the influence of reward sensitivity to predict bias was dependent on judgment context. Therefore, the predictive strength of positive motivational traits on bias depend on the judgment context in which the beliefs develop.

### Domain-*b*ased Bias Independent of Judgment Context

With respect to the first finding, the current study replicated findings from previous studies showing relatively greater optimism for risky choice options in the gain domain and pessimism in the loss domain (Kuhnen, 2015; Kuhnen and Miu, 2017). Experiment 1 observed this domain-based bias in a positive judgment context, wherein participants were asked to predict the probability that a risky stock was the “good” option relative to a safe bond. This result is consistent with biases observed in investor behavior (Todorov and Bollerslev, 2010; Bollerslev and Todorov, 2011; Daszyńska-Żygadło et al., 2014; Guiso et al., 2018), consumer spending habits (Millet et al., 2012), and private investing (Kuhnen and Miu, 2017). A key contribution of our study is that we extend these findings to negative judgment contexts, in which decision makers assess risky options based on their negative attributes. This manipulation provides important insights into the similarities and differences in cognitive processing when decision makers focus on positive versus negative choice attributes. Specifically, in Experiment 2, we observed a similar domain-based bias in beliefs when participants were asked to predict the probability that a risky stock was the “bad” option relative to a no-risk bond. These findings indicate that optimism for risky options in the gain domain and pessimism of risky options in the loss domain persist across positive and negative judgment contexts.

### Trait-*l*evel Reward Sensitivity Consistent Predictor of Beliefs and Behavior

Our second finding indicates that reward sensitivity was the most consistent predictor of beliefs. The limited findings of punishment sensitivity and beliefs suggest weak additional support for trait-level influences over affective state. Our results hold implications for previous studies indicating that levels of reward sensitivity are associated with differences in stock trading behavior. In particular, previous research indicates that after periods of increased or decreased stock market returns, those with high reward sensitivity prefer riskier portfolios and trade more often, but these behaviors do not increase profits (Muehlfeld et al., 2013). Our results suggest that the behavior observed by Muehlfeld et al. (2013) in reward-sensitive traders is driven by relatively greater optimism about stocks that yield positive dividends above and below the average investment return – particularly, when traders are focused on positive stock attributes (e.g., profits). Muehlfeld et al. (2013) also reported that highly punishment sensitive investors enhance their profits after positive shocks by increasing trading without overpaying for stocks. Likewise, our findings indicate no effect of punishment sensitivity on beliefs about stock outcomes in contexts that emphasize identifying profitable stocks (Experiment 1) or identifying poorer performing stocks (Experiment 2). Instead, this finding supports either a weak or non-existent relationship between punishment sensitivity and learning about punishment expectancies such as low performing stocks (Zinbarg and Mohlman, 1998). Collectively, our results suggest that individuals with high trait-levels of reward sensitivity are more likely to overestimate the value of risky options in the gain domain and underestimate the value of risky options in the loss domain, while punishment sensitivity is largely unrelated to the formation of beliefs. The specificity of reward sensitivity consistently driving effects could be due to the more stable consistency of the characteristic, or could relate to the properties of the trait itself- individuals more sensitive to reward may first orient to rewards (Baumeister et al., 2007) and then implement a strategy of maximizing rewards (Scheres and Sanfey, 2006), in this case, payouts. Study-specific findings underscore the role of contextual factors in determining the strength of which positive motivational traits influence beliefs about risky choices.

In line with our results for domain-based bias, reward sensitivity also emerged as a strong, consistent predictor of choice behavior regardless of judgment context, while negative affect was a less consistent predictor limited to the negative judgment context. Results for behavior indicated that, across judgment contexts, higher levels of reward sensitivity and negative affect were associated with poorer performance based on objective expected values. These findings compliment prior research showing that trait positivity predicts rejection rates for unfair offers in the Ultimatum Game (Dunn et al., 2010), in which rejecting unfair offers means forgoing potential financial gains. While this earlier study also found a relationship between NAS (anger) and rejection rates, the influence of trait positivity was not explained by fluctuations in negative affect; each had a separate impact. Our findings similarly suggest relatively independent effects of positive motivational traits and negative affect states on choice behavior as the former was more consistently associated with probability estimation errors. Theory and research have suggested that negative emotions can lead to suboptimal risk-taking behavior by increasing the salience of potential losses (Bordalo et al., 2012; Guiso et al., 2018) or the curvature of the utility function (Loewenstein, 2000; Guiso et al., 2018). The current study provides support for the latter hypothesis, as high negative affect predicted suboptimal choice behavior across gain and loss domains. Our results further suggest that the impact of negative emotional states on choice performance is independent of effects on risk preferences as we found no relationship between personal characteristics and stock selection rates on trials with 50% objective probabilities and no prior payout history (i.e., first trial of each block).

### Trait-*l*evel Reward Responsiveness and Inhibition Differentially Predict Biases in Gain and Loss Contexts

Lastly, we found that the relationship between positive motivational traits and domain-specific biases in belief depended on the congruence of trait and judgment context valences. Specifically, our results indicate that high reward sensitivity predicts greater bias and suboptimal choice behavior in positive judgment contexts, yet only predictive of suboptimal choice behavior in negative judgment contexts. NAS is also only predictive of suboptimal behavior in negative judgment context. These findings extend those of previous reports indicating that perceptions of wealth are enhanced in contexts that emphasize economic growth while perceptions of poverty are enhanced in contexts that emphasize scarcity (Millet et al., 2012). Specifically, our results indicate that context effects on perceptions about economic choices and conditions depend on one’s reward sensitivity and NAS. Observed judgment context effects may also extend earlier findings indicating that during deterministic learning, outcome expectancies for gain-associated cues are predicted by levels of reward responsiveness but not levels of punishment sensitivity (Zinbarg and Mohlman, 1998), as this earlier study did not control the expectancy-task’s judgment context. Combined findings from previous literature and the current study therefore underscore the role of judgment context valence in shaping the influence of positive behavioral motivations on economic beliefs.

### Limitations and Future Directions

The main findings of this study highlight the influence of motivational traits and decision contexts on the formation of biased beliefs about economic choice options. One limitation of the present study is that our study relied on behavioral measures of risk-taking, which have been noted to have poor reliability (Frey et al., 2017). While our findings may have been strengthened by the addition of other measures, the primary focus of our research was on belief formation. Therefore, our findings contribute to the literature especially regarding effects of personal characteristics on beliefs. Furthermore, some individual difference measures did not cover the entire range of possible responses for our scales, range of personalities, affective states, and only included undergraduate students. As such, future studies may rely on pre-screening participants to obtain samples that fully represent the scales, range of personal characteristics, and population for analysis. In addition, the cross-sectional nature of the study limited our ability to examine possible influences of affective states on behavioral motivations, or vice-versa (Harlé and Sanfey, 2010; Chiew and Braver, 2011). This limitation may be addressed by incorporating additional testing intervals so that relationships between states and traits can be examined over time. Finally, while our study represents an important step in determining factors that influence economic decision making in different decision contexts, we only compared effects of valenced motivational traits and affective states. Future studies may expand on this area of inquiry by considering other personal characteristics such as sense of power, self-worth, or emotional intelligence that have also been linked with decision biases (Anderson and Galinsky, 2006; Dunn et al., 2010; Panno et al., 2015).

Despite these limitations, the present experiments provide important insights into factors that may influence economic decision making across decision domains that yield gains versus losses, and decision contexts that emphasize identifying positive versus negative choice options. Our findings indicate that trait-level differences in positive motivation predict domain-specific biases in belief about economic choice options. We also demonstrate that the valence of one’s decision context determines the strength of positive motivations to predict biased beliefs. And finally, we find that state-level differences also play a role in determining economic decision making, but their effects are generally weaker than those of motivational traits. Such findings hold implications for choice architecture, suggesting that interactions of personal characteristics and the valence of decision contexts should be taken into consideration. Doing so may help to improve outcomes for decision makers across a range of contexts that involve risky choices with the potential for gains (e.g., investment decisions) and losses (e.g., healthcare decisions).

## Ethics Statement

This study was carried out in accordance with the recommendations of the University of Central Florida’s Institutional Review Board with verbal informed consent from all subjects. The Institutional Review Board determined the study was minimal risk and therefore did not require written informed consent. All subjects gave verbal informed consent in accordance with the Declaration of Helsinki. This protocol was approved by the Institutional Review Board.

## Author Contributions

KF and NL conceived and designed the study, analyzed and interpreted the results, revised the manuscript, and read and approved the submitted version. KF collected the data and wrote the first draft of the manuscript.

## Conflict of Interest Statement

The authors declare that the research was conducted in the absence of any commercial or financial relationships that could be construed as a potential conflict of interest.
