# Supplementary material for: Reward Responsiveness and Inhibition Traits Differentially Predict Economic Biases in Gain and Loss Contexts
Source: Front Psychol. 2019 Aug 23;10:1948. doi: 10.3389/fpsyg.2019.01948 (PMC6716470; doi:10.3389/fpsyg.2019.01948)
Supplement: Supplementary file 1 [file Table_1.docx]

**Supplementary Materials**

***Investment Task Instructions***

Welcome to the Investment Study!

The purpose of the study is to understand how people make economic decisions when they have to learn about their options over time.

In this study you will work on an investment task. During the task, you will repeatedly invest in one of two securities: A risky security (i.e., a stock with risky payoffs) and a riskless security (i.e., a bond with a known payoff).

You will also be asked to provide estimates as to how good an investment in the stock is, after observing its payoffs.

In the task, there are two types of conditions you can face: the GAIN condition and the LOSS condition.

In the GAIN condition, the two securities will only provide POSITIVE payoffs. In the LOSS condition, the two securities will only provide NEGATIVE payoffs.

In the GAIN condition, on any trial, if you choose to invest in the bond, you get a payoff of $6 for sure at the end of the trial. If you choose to invest in the stock, you will receive a dividend which can be either $10 or $2.

The stock can either be good or bad, and this will determine the likelihood of its dividend being high or low. In the GAIN condition, if the stock is good then the probability of receiving the $10 dividend is 70% and the probability of receiving $2 is 30%.

The dividends paid by this stock are independent from trial to trial, but come from this exact distribution. In other words, once it is determined by the computer that the stock is good, then on each trial the odds of the dividend being $10 are 70%, and the odds of it being $2 are 30%.

Example


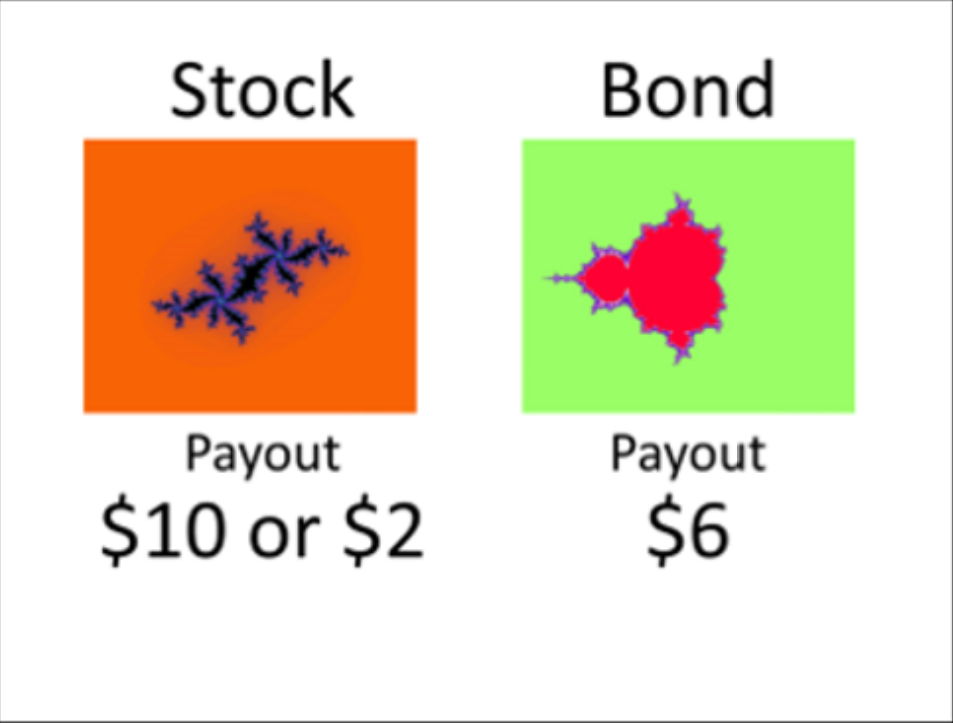


In the GAIN condition, if the stock is bad then the probability of receiving the $10 dividend is 30% and the probability of receiving the $2 dividend is 70%.

Once the computer determines that the stock is either good or bad, that is maintained on each trial within the block.

In the LOSS condition, on any trial, if you choose to invest in the bond, you get a payoff of -$6 for sure at the end of the trial. If you choose to invest in the stock, you will receive a dividend which can be either -$10 or -$2.

The stock can either be good or bad, and this will determine the likelihood of its dividend being high or low. In the LOSS condition, if the stock is good then the probability of receiving the -$10 dividend is 30% and the probability of receiving the -$2 dividend is 70%.

The dividends paid by this stock are independent from trial to trial, but come from this exact distribution. In other words, once it is determined by the computer that the stock is good, then on each trial the odds of the dividend being -$10 are 30%, and the odds of it being -$2 are 70%.

Example


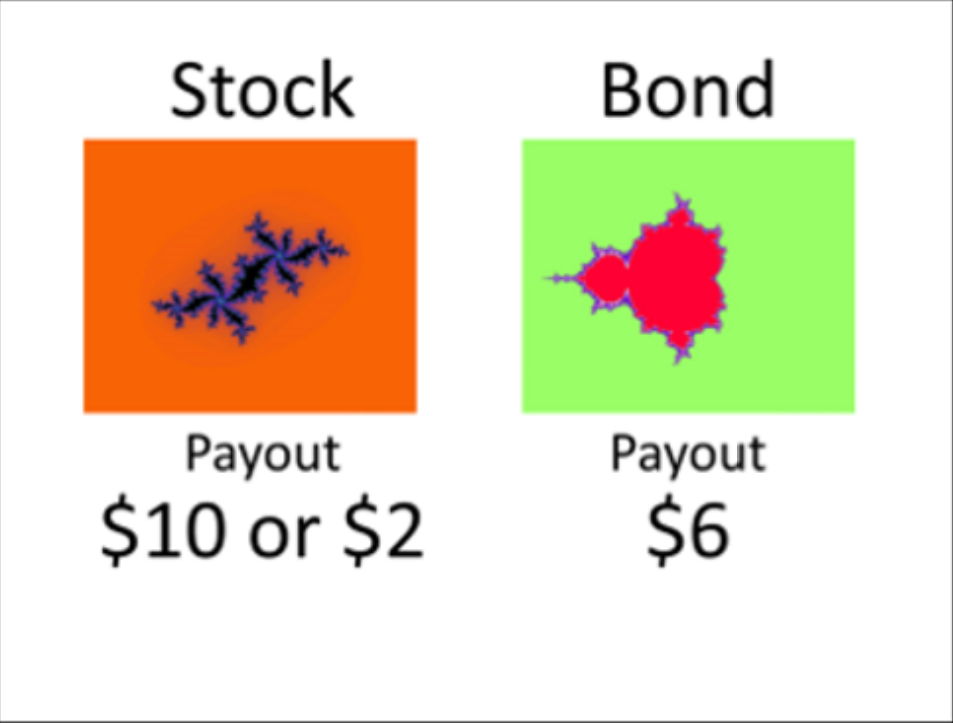


In the LOSS condition, if the stock is bad then the probability of receiving the -$10 dividend is 70% and the probability of receiving -$2 is 30%.

Again, once the computer determines that the stock is either good or bad, that is maintained on each trial within the block.

*Note, that these instructions are the same as the gain condition, except you can only lose money in the loss condition. In the loss condition, a “good” stock is probably not what you would first think. In this condition, the “good stock” is the one that loses the least amount of money. In other words, the stock that is more likely to lose only $2 is the good stock in the loss condition.

Choice Phase

At the beginning of each block of 6 trials, you will not know which type of stock the computer selected for that block. You may be facing a good stock or the bad stock, with equal probability. On each trial’s choice phase, you will decide whether you want to invest in the stock (and accumulate the dividend paid by the stock), or the bond (and add the sure-thing payoff to your earnings).

Example


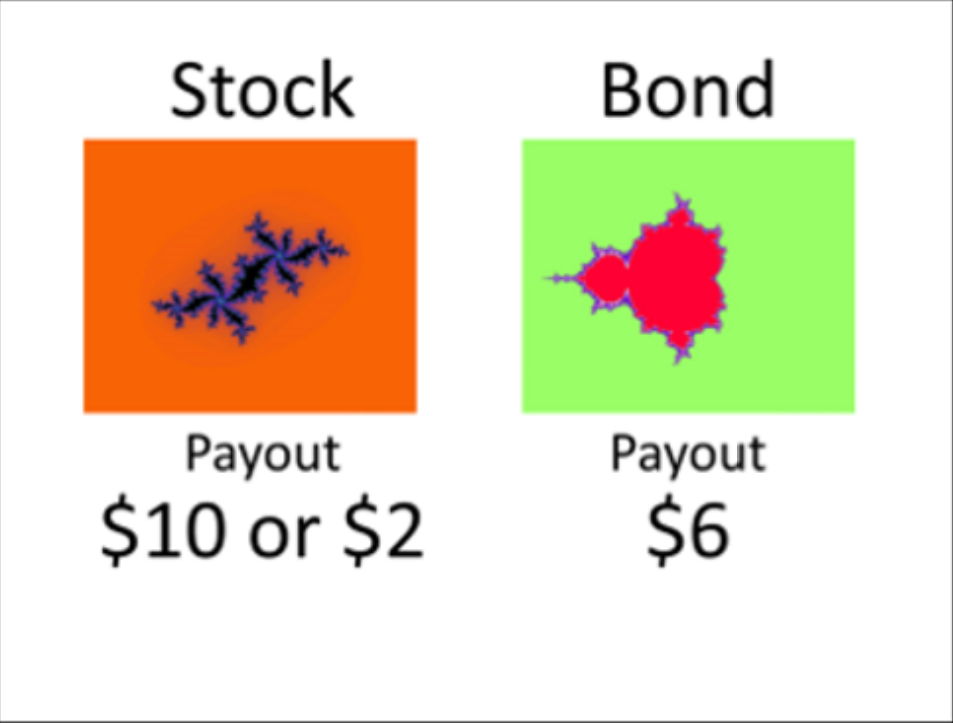


Stock Payout Phase

Then, a stockbroker will tell you the dividend amount paid by the stock – no matter if you chose the stock or the bond. To show us you’re paying attention to this phase, we ask that you tell us the gender of the stockbroker by making a button response.

Example


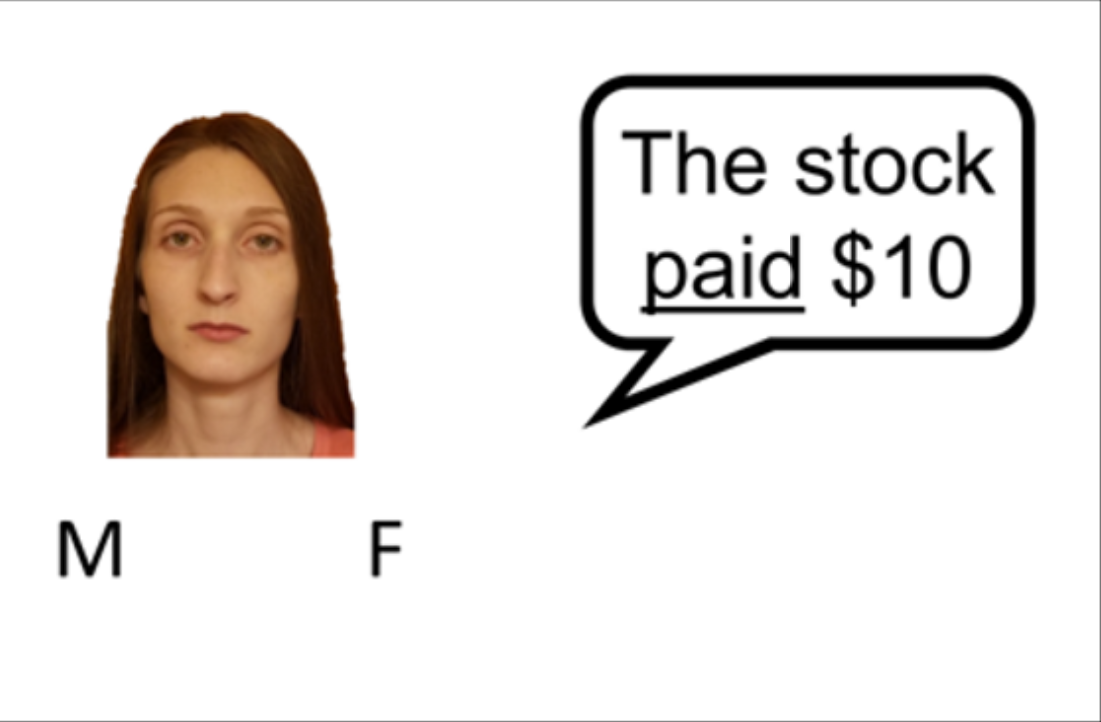


“**Note, that you will always be told the outcome of the stock regardless of whether you chose the bond or stock*.”

Good Stock Probability Estimation Phase

Then, we’ll ask you to estimate the probability that the current stock is a good one. Your answer must be a value from 0% to 100%.

*Remember that:

In the GAIN condition, a good stock gives $10 payouts 70% of the time and $2 payouts 30% of the time.

In the LOSS condition, a good stock gives -$2 payouts 70% of the time and -$10 payouts 30% of the time.

Example


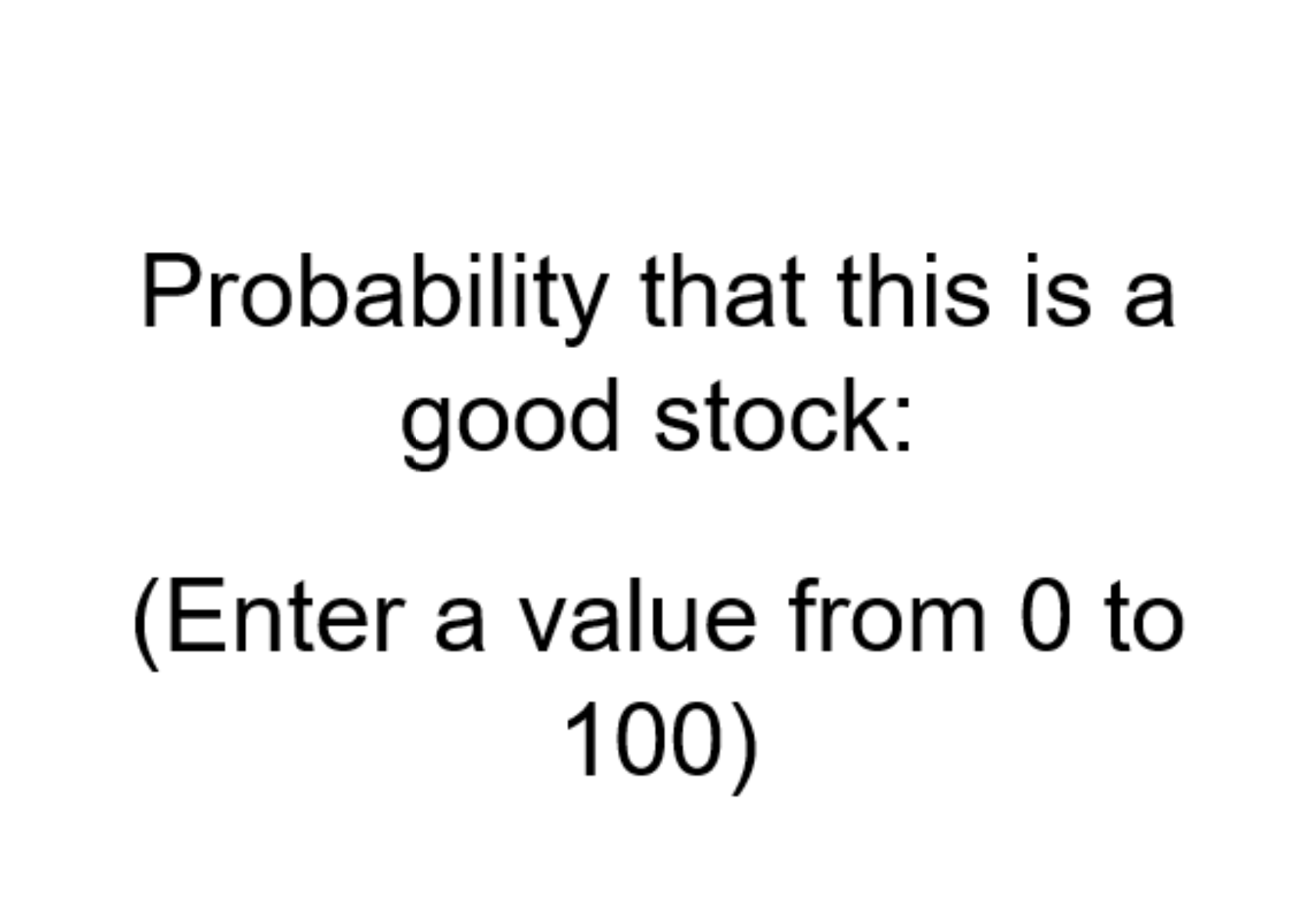


Estimation Confidence Phase

Finally, we want to know how much you trust your ability to come up with the correct probability estimate that the stock is good. In other words, we want to know how confident you are that the probability you estimated is correct. You’ll be asked to make a confidence rating on a 1-to-9 scale (1=lowest confidence, 9=highest confidence).

Example


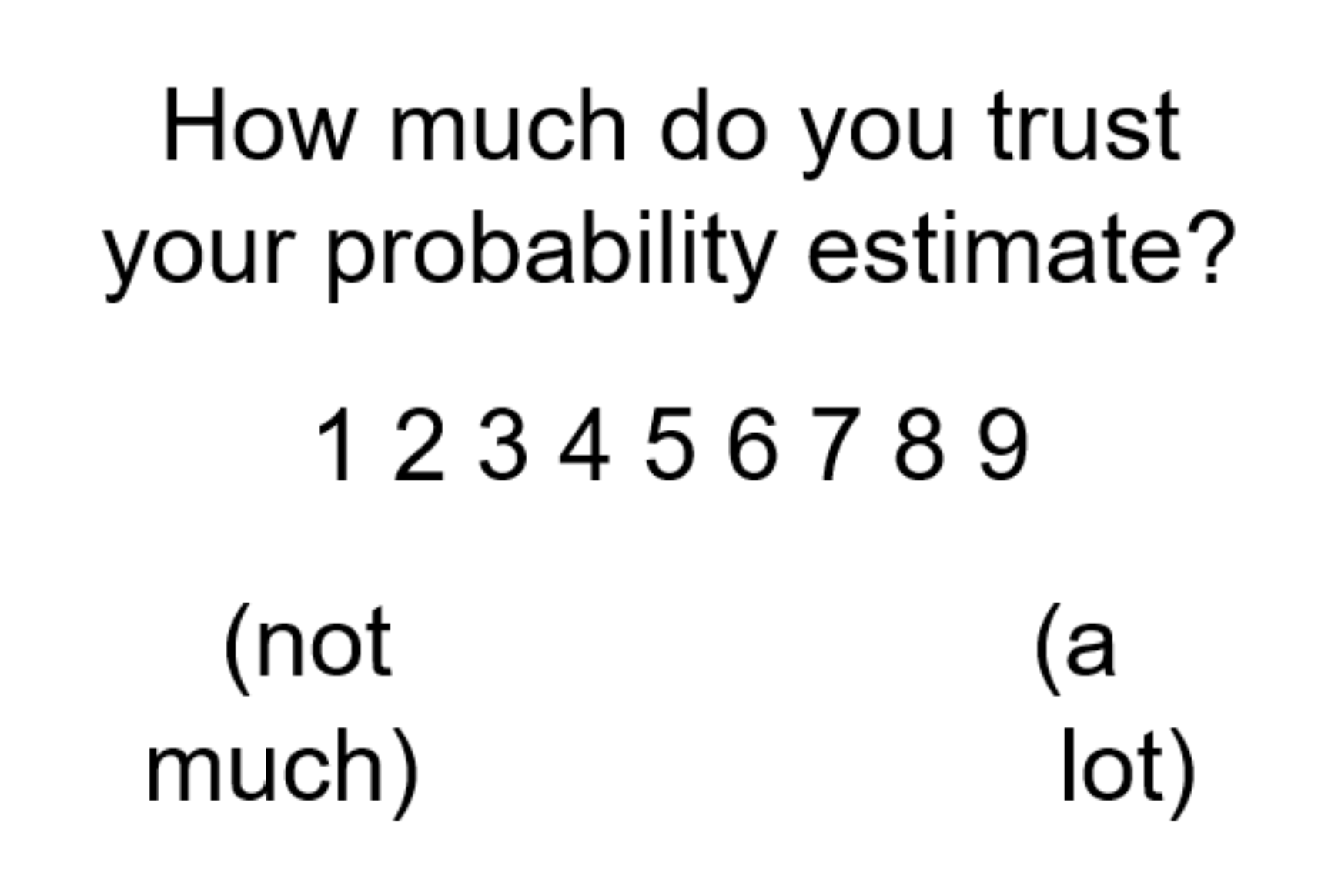


There is always an objective, correct probability that the stock is good, which depends on the history of dividends paid by the stock already. For instance, at the beginning of each block of trials, the probability that the stock is good is exactly 50%, and there is no doubt about this value.

As you observe the dividends paid by the stock you will update your belief whether it is good. How much you trust your ability to calculate this probability could vary. Sometimes you may not be too confident in the probability estimate you calculated and sometimes you may be highly confident in this estimate.

[Experimenter explained that each trial starts at 50-50, and that the participant is making the estimation after they see the result of the stock.]

Payment

From good stock probability estimates: We will pay you 10 cents for every probability estimate that is within 5% of the true probability. Earnings from accurate probability estimates will be added to your $15 base payment.

From investment choices: On each trial, you will see a running total of accumulated earnings from your investment choices. At the end of the study, we will add 10% of your final accumulated earnings to your base payment.

*Note, if you have a negative investment total, will we will subtract 10% of this amount from your base payment, but the minimum subject payment is $10.

[Experimenter would ask “Do you have any additional questions?”]

Consent note: The individual depicted in the “Example” task provided written informed consent for the publication of this image.

***Objective Probability Calculation***

For Experiment 1, the equation provides the Bayesian Posterior starting with a 50% to 50% prior that the stock is paying from the good distribution, and takes into account the history of payouts observed for that stock within a block. Each trial provides a stock payout observation (either an optimal “good” payout or a less desirable relatively “bad” payout) and the Bayesian Posterior is updated accordingly. Each block contains six trials, which means the Bayesian Posterior updates six times per block.

The objective Bayesian posterior for this experiment can be calculated from the following equation:

$$\frac{1}{1+\frac{1-p}{p}*{(\frac{q}{1-q})}^{n-2t}}$$

For Experiment 1, the objective Bayesian posterior that the stock on a given trial is a good one, is given by p = 50% for the prior the stock is good before any payouts are observed for the block, q = 70% for the probability that the stock will pay from the good distribution (provide a “good” payout) in each trial, n is the total trials observed thus far in the block, and t is the total number of “good” payouts.

For Experiment 2, the objective Bayesian posterior that the stock on a given trial is a bad one, is given by p = 50% for the prior the stock is good before any payouts are observed for the block, q = 30% for the probability that the stock will pay from the good distribution (provide a “good” payout) in each trial, n is the total trials observed thus far in the block, and t is the total number of “good” payouts.

Citation: Appendix B “Objective Bayesian Posterior Belief” from Kuhnen, C. M. (2015). Asymmetric learning from financial information. *Journal of Finance, 70*(5), 2029-2062.

***Supplementary Table 1.*** Investment Task Structure***.*** For each participant, the investment task included a total of 12 domain blocks with six trials included in each block. The following table only gives a potential order of both the predetermined and pseudorandomized domain blocks and of the stock payout outcomes within those blocks. Within each block, participants learn a new stock payout with each trial. In addition to the payout history updating, the objective probability that the stock “is good” based on the Bayesian Posterior values also updates (Experiment 1 example).

| Block | Domain | Trial Within Block | Stock Payout | Bayesian Posterior |
| --- | --- | --- | --- | --- |
| 1 | Loss | 1 | -$2 | 70.00% |
|  |  | 2 | -$10 | 50.00% |
|  |  | 3 | -$2 | 70.00% |
|  |  | 4 | -$2 | 84.48% |
|  |  | 5 | -$10 | 70.00% |
|  |  | 6 | -$2 | 84.48% |
| 2 | Loss | 1 | -$2 | 70.00% |
|  |  | 2 | -$2 | 84.48% |
|  |  | 3 | -$10 | 70.00% |
|  |  | 4 | -$2 | 84.48% |
|  |  | 5 | -$2 | 92.70% |
|  |  | 6 | -$2 | 96.74% |
| **.** | **.** | **.** | **.** | **.** |
| **.** | **.** | **.** | **.** | **.** |
| **.** | **.** | **.** | **.** | **.** |
| 12 | Gain | 1 | $2 | 30.00% |
|  |  | 2 | $2 | 15.52% |
|  |  | 3 | $2 | 7.30% |
|  |  | 4 | $2 | 3.26% |
|  |  | 5 | $2 | 1.43% |
|  |  | 6 | $2 | 0.62% |

***Supplementary Table 2.*** Rank Correlations. Spearman’s rho correlations for personal characteristics and outcome measures in both Experiment 1 and Experiment 2.

|  | 1 | 2 | 3 | 4 | 5 | 6 | 7 | 8 | 9 | 10 | 11 | 12 | 13 |
| --- | --- | --- | --- | --- | --- | --- | --- | --- | --- | --- | --- | --- | --- |
| Exp. 1 |  |  |  |  |  |  |  |  |  |  |  |  |  |
| 1. BAS-RR | — |  |  |  |  |  |  |  |  |  |  |  |  |
| 2. BIS | 0.17 | — |  |  |  |  |  |  |  |  |  |  |  |
| 3. PAS | **.28**** | -0.15 | — |  |  |  |  |  |  |  |  |  |  |
| 4. NAS | -0.08 | 0.08 | -0.03 | — |  |  |  |  |  |  |  |  |  |
| 5. Overall Valenced Error | **.24*** | 0.04 | 0.19 | 0.07 | — |  |  |  |  |  |  |  |  |
| 6. Gain Valenced Error | 0.16 | -0.06 | **.232*** | 0.16 | **.67**** | — |  |  |  |  |  |  |  |
| 7. Loss Valenced Error | -0.08 | -0.14 | -0.02 | 0.09 | **-.40**** | **.31**** | —- |  |  |  |  |  |  |
| 8. Gain Optimal Choice | **-.30**** | 0.00 | 0.03 | -0.03 | -0.01 | -0.06 | -0.03 | — |  |  |  |  |  |
| 9. Loss Optimal Choice | **-.21*** | 0.11 | 0.09 | -0.04 | 0.09 | 0.03 | -0.10 | **.75**** | — |  |  |  |  |
| 10. Total Optimal Choice | **-.28**** | 0.06 | 0.07 | -0.04 | 0.04 | -0.01 | -0.07 | **.94**** | **.93**** | —- |  |  |  |
| 11. Gain Risk Taking | 0.15 | 0.06 | 0.11 | -0.07 | -0.06 | -0.05 | 0.02 | -0.19 | -0.19 | **-.22*** | — |  |  |
| 12. Loss Risk Taking | -0.08 | 0.11 | 0.08 | -0.03 | -0.03 | -0.11 | 0.02 | -0.07 | -0.11 | -0.09 | **.29**** | — |  |
| 13. Total Risk Taking | 0.06 | 0.12 | 0.16 | -0.06 | -0.02 | -0.04 | 0.04 | -0.18 | -0.18 | -0.20 | **.82**** | **.77**** | — |
| Exp. 2 |  |  |  |  |  |  |  |  |  |  |  |  |  |
| 1. BAS-RR | — |  |  |  |  |  |  |  |  |  |  |  |  |
| 2. BIS | 0.18 | — |  |  |  |  |  |  |  |  |  |  |  |
| 3. PAS | **.21*** | -0.16 | — |  |  |  |  |  |  |  |  |  |  |
| 4. NAS | 0.18 | **.31**** | 0.07 | — |  |  |  |  |  |  |  |  |  |
| 5. Overall Valenced Error | 0.10 | **.24*** | 0.03 | 0.08 | — |  |  |  |  |  |  |  |  |
| 6. Gain Valenced Error | 0.15 | 0.09 | 0.11 | -0.07 | **.69**** | — |  |  |  |  |  |  |  |
| 7. Loss Valenced Error | 0.03 | -0.16 | 0.11 | -0.13 | **-.60**** | 0.05 | —- |  |  |  |  |  |  |
| 8. Gain Optimal Choice | **-.29**** | -0.14 | **-.22*** | **-.25**** | -0.15 | -0.03 | 0.15 | — |  |  |  |  |  |
| 9. Loss Optimal Choice | -0.18 | 0.01 | -0.05 | **-.22*** | -0.05 | 0.00 | 0.05 | **.66**** | — |  |  |  |  |
| 10. Total Optimal Choice | **-.26**** | -0.07 | -0.16 | **-.26**** | -0.12 | -0.04 | 0.09 | **.92**** | **.90**** | —- |  |  |  |
| 11. Gain Risk Taking | 0.06 | 0.15 | -0.08 | 0.04 | -0.05 | -0.11 | -0.16 | -0.06 | -0.08 | -0.06 | — |  |  |
| 12. Loss Risk Taking | 0.04 | 0.12 | -0.16 | -0.01 | -0.05 | -0.13 | -0.06 | 0.01 | 0.02 | 0.02 | **.48**** | — |  |
| 13. Total Risk Taking | 0.04 | 0.15 | -0.13 | 0.01 | -0.06 | -0.16 | -0.15 | -0.04 | -0.06 | -0.04 | **.88**** | **.83**** | — |

***Supplementary Table 3.*** Tests of valence estimation error bias in Experiment 1. One-sample t-tests of estimation error bias.

|  | Test Value = 0 | | | | | |
| --- | --- | --- | --- | --- | --- | --- |
|  | t | df | Sig. (2-tailed) | Mean Difference | 95% Confidence Interval of the Difference | |
|  |  |  |  |  | Lower | Upper |
| Overall Estimation Error | 6.091 | 88 | .000** | 0.048 | 0.033 | 0.064 |
| Gain Domain Error | 4.245 | 88 | .000** | 0.032 | 0.017 | 0.047 |
| Loss Domain Error | -2.406 | 88 | .018* | -0.017 | -0.030 | -0.003 |

**Notes significance at *p* < .001, while *notes significance at *p* < .05.

***Supplementary Table 4***. Tests of valence estimation error bias in Experiment 2. One-sample t-tests of estimation error bias.

|  | Test Value = 0 | | | | | |
| --- | --- | --- | --- | --- | --- | --- |
|  | t | df | Sig. (2-tailed) | Mean Difference | 95% Confidence Interval of the Difference | |
|  |  |  |  |  | Lower | Upper |
| Overall Estimation Error | 5.129 | 102 | .000** | 0.049 | 0.030 | 0.068 |
| Gain Domain Error | 1.841 | 102 | .069 | 0.014 | -0.001 | 0.029 |
| Loss Domain Error | -4.814 | 102 | .000** | -0.036 | -0.050 | -0.021 |

**Notes significance at *p* < .001, while *notes significance at *p* < .05.

***Supplementary Table 5.*** Rank correlations for the cross-studies analysis.

|  | 1 | 2 | 3 | 4 | 5 | 6 | 7 |
| --- | --- | --- | --- | --- | --- | --- | --- |
| 1. Exp. | — |  |  |  |  |  |  |
| 2. BAS-RR | 0.128 | — |  |  |  |  |  |
| 3. NAS | 0.087 | 0.077 | — |  |  |  |  |
| 4. Overall Valenced Error | -0.01 | .162* | 0.075 | — |  |  |  |
| 5. Total Optimal Choice | -0.024 | -.271** | -.150* | -0.056 | — |  |  |
| 6. Gain Optimal Choice | 0.001 | -.287** | -.143* | -0.093 | .927** | — |  |
| 7. Loss Optimal Choice | -0.043 | -.208** | -0.139 | 0.003 | .913** | .702** | — |

**Notes significance at *p* < .001, while *notes significance at *p* < .05.

***Supplementary Table 6.*** Model statistics for the multiple linear regressions in the Cross-studies analysis by dependent variable and then model type (trait or state); includes some assumptions testing information.

| Model | *F* | *df* | Adj R^2^ | Sig | Breusch- Pagan | Durbin-Watson |
| --- | --- | --- | --- | --- | --- | --- |
| Overall Error Trait | 1.887 | 3, 188 | 0.014 | 0.133 | .002 S | 2.042 |
| Overall Choice Trait | 4.824 | 3, 188 | 0.057 | 0.003 | .774 | 1.983 |
| Overall Choice State | 3.122 | 3, 188 | 0.032 | 0.027 | .585 | 2.168 |

Note: S indicates that the magnitude of heteroscedasticity is not large enough to correct for, while L indicates the magnitude should potentially be corrected for according to Cohen, Cohen, West, & Aiken (2007)

***Supplementary Table 7.*** Predictor variable information and collinearity statistics for the Cross-studies multiple linear regressions; includes dependent variable and then model type (trait or state).

|  |  |  |  |  |  | Collinearity Statistics | |
| --- | --- | --- | --- | --- | --- | --- | --- |
| Model | Predictor | B | β | t | Sig. | Tolerance | VIF |
| Overall Error Trait | Exp. | -0.053 | -0.604 | -0.755 | 0.451 | 0.008 | 123.728 |
|  | BAS-RR | 0.008 | 0.158 | 2.166 | 0.032 | 0.972 | 1.029 |
|  | Interaction | 0.003 | 0.624 | 0.782 | 0.435 | 0.008 | 123.425 |
| Total Choice Trait | Exp. | -0.044 | -0.269 | -0.344 | 0.732 | 0.008 | 123.728 |
|  | BAS-RR | -0.027 | -0.271 | -3.798 | 0.000 | 0.972 | 1.029 |
|  | Interaction | 0.002 | 0.246 | 0.315 | 0.753 | 0.008 | 123.425 |
| Total Trait State | Exp. | -0.081 | -0.492 | -1.722 | 0.087 | 0.062 | 16.099 |
|  | NAS | -0.005 | -0.108 | -1.297 | 0.196 | 0.73 | 1.369 |
|  | Interaction | 0.006 | 0.511 | 1.736 | 0.084 | 0.058 | 17.101 |
